# Stigma in people living with bipolar disorder and their families: a systematic review

**DOI:** 10.1186/s40345-023-00290-y

**Published:** 2023-02-20

**Authors:** Maryam Latifian, Kianoush Abdi, Ghoncheh Raheb, Sheikh Mohammed Shariful Islam, Rosa Alikhani

**Affiliations:** 1 Psychosis Research Center, University of Social Welfare and Rehabilitation Sciences, Tehran, Iran; 2 Department of Rehabilitation Management, University of Social Welfare and Rehabilitation Sciences, Tehran, Iran; 3 Institute for Physical Activity and Nutrition, School of Exercise and Nutrition Sciences, Deakin University, Melbourne, Australia

**Keywords:** Stigma, Bipolar Disorder, Mental Health, Family, Systematic Review

## Abstract

**Background:**

Stigma affects different life aspects in people living with bipolar disorder and their families. This study aimed to examining the experience of stigma and evaluating predictors, consequences and strategies to combat stigma in people with bipolar disorder and their families.

**Methods:**

We conducted a systematic review according to the Preferred Reporting Items for Systematic reviews and Meta-Analyses (PRISMA) in 2022. We extensively reviewed six online databases (PubMed, Scopus, Medline, EMBASE, Web of Science and Google Scholar). Articles published in the English language about stigma in people living with bipolar disorders and their families were included.

**Results:**

A total of 42,763 articles were retrieved, of which 40 articles from 14 countries were included in this study (n = 7417 participants). Of the 40 articles, 29 adopted quantitative methods (72.5%), two used mixed-methods (5%), eight used qualitative (20%) methods, and one was a case series (2.5%). The results of the studies were categorized into four themes: 1. Stigma experienced by people living with bipolar disorders and their families, 2. Predictors of stigma in people living with bipolar disorders and their families, 3. Consequences of stigma in people living with bipolar disorders and their families, 4. Effective interventions and strategies to reduce stigma in people living with bipolar disorders and their families.

**Conclusion:**

The results of this study might be useful to design psychiatric cognitive interventions to reduce stigma in people living with bipolar disorders and their families and designing community-based interventions to normalize bipolar disorder at the community level.

## Background

Psychiatric disorders are one of the five leading causes of disability. Bipolar disorder is one of the most persistent, important, and severe psychiatric disorders. Approximately 1% of the population suffers from bipolar disorders and is equally common in both genders (Angst 2013; Jolfaei et al. 2019; Mohammadi et al. 2019). Bipolar disorder is described in the DSM-5 as a group of brain disorders that cause extreme fluctuation in mood, energy, and function, ranging from depressive to schizophrenia. People with bipolar disorder experience periods of excitement, overactivity, delirium, and euphoria (known as mania), and other periods of feeling sadness and hopelessness (known as depression) (Kaltenboeck et al. 2016; Khaleghi et al. 2018).

People with bipolar disorder have been neglected in the society. Several factors contribute to poor support for people with bipolar disorders, including costly care in medical centers, poor cooperation between supportive organizations and insurance companies, and de-institutionalization movement policies. As a result, most families are responsible for caring for people with bipolar disorders (BİLİR, 2018; Ghai et al. 2013). The experience of caring for such people can be different from other mental illnesses. The reason for this difference goes back to the nature of the disease, which has a periodic state and has an oscillating nature (Bruni et al. 2018). Stigma is one of the most common and challenging social issues that affect people living with bipolar disorder (Grover et al. 2019; Sharma et al., 2017).

Stigma can be considered a combination of three problems: lack of knowledge (ignorance and misinformation), negative attitudes (prejudice), and rejection or avoidance behaviors (discrimination) (Goffman 2003; Henderson et al. 2013). The history of stigma and rejection from society backs to ancient times (Gur et al. 2012). The presence of stigma is the most serious concern for patients; because they must cope with the disease and symptoms and adapt to negative attitudes and society labeling (Knight et al. 2006; Sibitz et al. 2011; Wong et al. 2009). Stigma impairs people’s quality of life and leads to their isolation and rejection of interpersonal relationships. The rejection from society and low self-esteem, following the fear of rejection, weakens living conditions, reduces income, and causes unemployment for people with bipolar disorders (Connell et al. 2014; Latifian et al. 2022a, b, c).

Stigma is also common among families of people living with bipolar disorder. The parents of such patients, reprimanding by ordinary and professional people, look for the causes of the disease and experience issues such as guilt attribution and social exclusion due to having a family member with such disorder (Koschorke et al. 2014; Latifian et al. 2022a, b, c; McCann et al. 2011). Family stigma contributes to decreased self-esteem, sleep disorders, decreased psychological well-being, and reduced quality of life (Wong et al. 2009). Another consequence of stigma is the inability of families to seek treatment. About 50–60% of people living with neurological disorders refuse treatment or care due to fear of stigma for themselves and their family members (Park & Park 2014). In the study by Ando et al., one of the reasons for the delay in counseling was concerned about people’s thoughts (Ando et al. 2013). In addition, stigma increases the risk of suicide in people living with bipolar disorders and their families and is cited as one of its causes (Aggarwal et al. 2014).

Although stigma is a global phenomenon, the experience of dealing with it and the discrimination varies across countries and even cities. In general, the reaction of people in the community to patients with psychiatric disorders can vary depending on the severity of illness, culture, and changes over time (Keshavarz et al. 2018; Shamsaiee et al., 2013).

The increasing experience of stigma in people living with bipolar disorders and their families, as well as the significant role of psychological and social interventions in reducing the stigma of this disease, make it more necessary to pay attention to the various dimensions of this phenomenon (Latifian et al. 2022a, b, c). So far, a number of review articles have been done in the field of stigma in bipolar disorder. For example, in the study by Perich et al., stigma in bipolar disorder is compared with schizophrenia, personality disorders and anxiety disorders. Also, general stigma has been compared with greater functional impairment and lower levels of functional impairment (Perich et al. 2022). In the study by Pal et al., only the researches that have been conducted in India in a quantitative and qualitative manner have been examined (Pal et al. 2021). Ellison et al. also conducted a review study. This review aimed to identify publications which investigated public attitudes and/or beliefs about bipolar disorder or explored internalised stigma in bipolar disorder between 1992 and 2012 (Ellison et al. 2013). In 2013, a review article was prepared by Hawke et al. The purpose of this article was to identify guidelines and specialized interventions for the development of stigma reduction initiatives in bipolar patients by April 2012 (Hawke et al. 2013).

Review of studies showed that each of the studies conducted in this field investigated one of the dimensions of stigma in one of the two groups of people living with bipolar disorders and their families. Even in some cases, differences and disagreements were seen in the results of these studies. Information on the different dimensions of stigma and blame and analyzing the similarities and differences of the results of previous studies in both groups, with a comprehensive review of this issue, can lead to a better and more comprehensive understanding of this issue. However, there are no systematic reviews addressing these issues. Therefore, the present study aimed at examining the experience of stigma and evaluating the predictors, consequences, and strategies to combat stigma in people living with bipolar disorder and their families by December 2022.

## Methods

We conducted a systematic review to analyze research findings on stigma in people living with bipolar disorders and their families. This study was performed according to the directions denoted by the Preferred Reporting Items for Systematic Reviews and Meta-Analyses (PRISMA) in 2022.

### Search strategy

This study included all articles published in English from the earliest indexed articles to December 2022, including descriptive, survey, correlation, case report, cohort, experimental, quasi-experimental, qualitative, and mixed-method studies on stigma in people living with bipolar disorders and their families. For this purpose, the keywords of "bipolar disorder"[Title/Abstract] OR "manic"[Title/Abstract] OR "depression"[Title/Abstract] AND "stigma"[Title/Abstract] OR "self-stigma"[Title/Abstract] OR "public stigma"[Title/Abstract] OR "internalized stigma"[Title/Abstract] OR "mental illness stigma"[Title/Abstract] OR "stigmatization"[Title/Abstract] AND "Family" [Title/Abstract] OR "caregiver"[Title/Abstract] were searched in the "PubMed", "Scopus", "Medline", "EMBASE", "Web of Science" and "Google Scholar" databases.

### Inclusion and exclusion criteria

The inclusion criteria were: articles published in English and presence of stigma in people living with bipolar disorder or their families. Exclusion criteria comprised the unavailability of full text, being a review study, and the lack of a clear explanation of research methods. Considering the statistical population of the research, the data obtained from the studies were categorized in terms of the subject, methodology, and validity and then analyzed. Before conducting the literature review, a form was prepared according to study goals and presented to the research team for data extraction after teaching them how to complete it.

### Quality assessment of screened studies

Three researchers independently performed a literature search in different databases, initial assessment of articles, qualifying articles, and checking their compliance with inclusion and exclusion criteria. In the case of disagreement, the consensus was reached with the help of a fourth researcher. Methodological quality of selected articles was assessed using the Critical Appraisal Skills Programmed (CASP). This instrument includes 12 questions about diagnostic tests (Singh 2013). Studied articles were divided into 3 categories (high quality, moderate quality and low quality). Those articles that were studied by CASP criteria and were categorized as moderate or high quality were used in this systematic review. All 40 remained articles were scored as moderate or high quality.

## Results

**Fig. 1:**
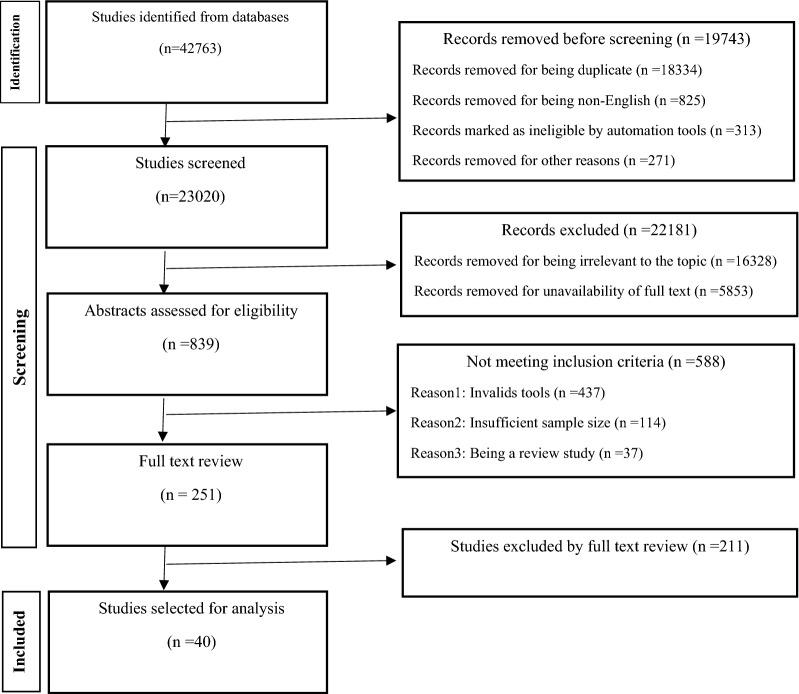
Summary of process used to identify and select studies for the review

In the first stage, after searching in different databases 42,763 records identified. In the second stage, 19,743 records, due to being duplicate or non-English language were removed. In the third stage, the records were screened and the titles of the articles were reviewed. After assessing the articles in terms of design and quality criteria by four experts in the fields of social work, psychology, and rehabilitation, the articles that had titles unrelated to the topic or did not have full text were removed and 839 articles that met the least scientific requirements for being included in a systematic review were chosen. In the fourth stage, after reviewing the abstracts of articles, several articles unqualified for being included in the study were omitted, leaving 251 articles. Some of the reasons for removing these articles at this stage were: lack of valid tools, insufficient sample size and being a review study. Then, in the last stage, the full text of 251 articles was reviewed and methodology was analyzed to provide an accurate definition and explanation of the target group, study design, sampling method, sample size, and the validity and reliability of data collection tools. Finally, 40 articles remained for final evaluation. Figure 1 shows the process of the inclusion and exclusion of primary studies until reaching the final synthesis.

After searching and qualitative evaluation of the studies during a systematic review, in the end, the final synthesis was performed on 40 articles. As it turns out in Table 1, quantitative research has accounted for a larger share of studies.

**Table 1:** Frequency distribution of articles by research method

	Research method	Number	Percent
1	Mix method	2	5
2	Qualitative Study	8	20
3	Case series	1	2.5
	Quantitative study		
4	Cross sectional	23	57.5
5	Comparative	2	5
6	Cohort	1	2.5
7	Quasi-experimental	3	7.5

According to Table 2, the distribution of studies in different countries is also easily understood. About 14 countries have directly studied this phenomenon, and some countries have conducted joint studies.

**Table 2:** Frequency distribution of articles according to the place of research

	Country	Number	Percent
1	India	7	17.5
2	Canada	5	12.5
3	USA	4	10
4	UK	4	10
5	IRAN	6	15
6	Brazil	2	5
7	Ethiopia	2	5
8	Turkey	2	5
9	Switzerland	3	7.5
10	China	1	2.5
11	Greece	1	2.5
12	Denmark	1	2.5
13	Austria	1	2.5
14	Argentina	1	2.5

The results of the studies were categorized into four themes: 1. Stigma experienced by people living with bipolar disorders and their families, 2. Predictors of stigma in people living with bipolar disorders and their families, 3. Consequences of stigma in people living with bipolar disorders and their families, 4. Effective interventions and strategies to reduce stigma in people living with bipolar disorders and their families.

### Stigma experienced by people living with bipolar disorders and their families

According to studies, people living with bipolar disorders and their primary caregivers and family members experience stigma. These people are easily labeled by the society and experience stigma as a result. In addition, the public blames family members for incompetence on the patient, leading to the onset or recurrence of the disease. As a result, family members avoid attending social events and try to hide the affected member from society as much as possible. In Table 3, it is mentioned information on articles that have examined the stigma experienced by people living with bipolar disorders and their families and a summary of the findings of these articles is reported.

**Table 3:** Information on articles that have examined the stigma experienced by people living with bipolar disorders and their families

	Author, Year,	Location of study	Study type	Sampling method	Sample size	Tools	Finding
1	Latifian et al. 2022a, b, c	Iran	Qualitative Method (Grounded theory)	Purposive sampling	20 family members of bipolar patients 4 bipolar patients 3 mental health professional	Qualitative interviews	Families of people living with bipolar disorder experience social deprivation, social isolation, and social rejection, which have irreparable consequences for them. This people being labelled by the community members and are socially deprived in many aspects of their lives. Consequently, to return to normal life, they are forced to accept the situation and try to adopt a method of indifference and distance themselves from other people as much as possible to face people less
2	Richard-Lepouriel et al., 2022	Switzerland	Qualitative Method	Purposive sampling	21 family members of bipolar patients	Qualitative interviews	People living with bipolar disorders and their families experienced stigma in five different dimensions, including language, identity, emotions, others’ reactions, and coping with bipolar disorder, highlighting the importance of specialized interventions to reduce stigma in this people
3	Grover et al., 2019	India	Cross-sectional study	Purposive sampling	103 caregivers of patients with bipolar disorder	Perceived discrimination Devaluation Scale (PDD)	People living with bipolar and their caregivers experience considerable dependence and stigma and there is a positive and significant correlation between more stigma and lower-income, as well as less emotional support in these individuals
4	Sharma et al., 2017	India	Cross-sectional study	Random sampling	Caregivers of 88 patients with schizophrenia and bipolar disorder	The Stigma Scale (SS)	Care burden for the family member of people living with bipolar disorder is mainly felt in leisure time, during communication with others, as well as in financial issues There was no significant difference in the experience of stigma between families with different socio-economic conditions
5	Pal et al., 2017	India	Cross-sectional study	Random sampling	123 participant (60 patients with BPAD, 33 patients with schizophrenia and 30 patients with anxiety disorders)	Rosenberg Self-Esteem Scale (RSES) Participation scale (PS) World Health Organization Quality Of Life—Brief Version – Hindi (WHOQOL-brief)	Bipolar patients experience substantial stigma and internalized stigma has significant impact on self-esteem, socio-occupational participation and functioning, and quality of life in this people
6	Bassirnia et al. 2015	USA	Cross-sectional study	Random sampling	112 patients with bipolar disorder and 112 corresponding treatment partners	Rosenberg Self-Esteem Scale (RSES)	Both people living with bipolar disorders and their treatment counterparts experience internal stigma There was a positive and significant relationship between internal stigma and introversion personality trait

### Predictors of stigma in people living with bipolar disorders and their families

The analysis of studies showed that several factors could pave the path for stigma in people living with bipolar disorders and their families. These factors are known as the predictors of stigma, which can either alone or in combination with each other create stigma. In Table 4, it is mentioned information on articles that have examined the predictors of stigma in people living with bipolar disorders and their families and a summary of the findings of these articles is reported.

**Table 4:** Information on articles that have examined the predictors of stigma in people living with bipolar disorders and their families

	Author, Year,	Location of study	Study type	Sampling method	Sample size	Tools	Finding
1	Favre et al. 2022	Switzerland	Qualitative Method	Purposive sampling	22 bipolar patients	Qualitative interviews	Items such as lack of information/knowledge, labeling, generalization, banalization and reaction induced by public stigma in the general population, or among professionals such as health care professionals and employers were identified as predictors of stigma in bipolar patients
2	Shumet et al. 2021	Ethiopia	Cross-sectional study	Systematic random sampling	418 bipolar patients	Stigma Scale (DISC) 12	About 24.9% of people living with bipolar disorder had internal stigma based on the ISMI scale, which was significantly associated with unemployment, low education, low self-esteem, poor social support, and more than three times hospitalization
3	Pal 2020	India	Cross-sectional study	Purposive sampling	75 bipolar patients	The Internalized Stigma of Mental Illness (ISMI) scale	Internal stigma in people living with bipolar disorders was significantly associated with factors such as monthly income, job and social performance, and education, and the fact that internal stigma significantly influenced the self-esteem, participation, and quality of life of people living with bipolar disorders
4	Engidaw et al. 2020	Ethiopia	Cross-sectional study	Systematic random sampling	418 bipolar patients	Quality of Life scale (WHO)	Tolerance to stigma was low in people living with bipolar disorder, which could be related to inadequate education and unemployment in these patients. Having academic education and a suitable job can boost self-esteem in these patients and their families, increasing their self-satisfaction and causing them to become less affected by others’ judgments and prejudices
5	Clemente et al. 2017	Brazil	Qualitative Method (Ethnography)	Purposive sampling	23 psychiatrists	Qualitative interviews Rosenberg Self-Esteem Scale (RSES)	People living with bipolar disorders experience less stigma due to the individualistic culture in developed societies. This is while stronger interactions and cohesion between people in underdeveloped societies can essentially enhance stigma in these patients
6	Howland et al. 2016	USA	Mixed Method	Available sampling	115 bipolar patients in quantitative phase 21 bipolar patients in the qualitative phase	Mental Illness (ISMI) scale General Self-Efficacy (GSE) Scale Brief Psychiatric Rating Scale (BPRS) Montgomery–Asberg Depression Rating Scale (MADRS) Young Mania Rating Scale (YMRS) Qualitative interviews	Internal stigma and self-efficacy were associated with each other, and internal stigma was also associated with depression symptoms such as anxiety, as well as feeling guilty and suspicious in people living with bipolar disorder
7	Ellison et al., 2015	UK	Cross-sectional study	Snowballing sampling	753 members of the UK population (Using an online questionnaire distributed via email, social networking sites and public institutions)	Clinicians’ Attitudes Scale Version 4	The prevalence of stigma was between 37 and 57% in people living with bipolar disorders and their families. In addition, it was noted that stigma in people living with bipolar disorders was influenced by hospitalization in psychiatric hospitals and the ability to work, and psychosocial factors and emotional attitudes were reported as two important factors in labeling
8	Bonnington et al., 2014	UK	Qualitative Method	Purposive sampling	29 bipolar patients	Qualitative interviews	Social and cultural structures influence the atmosphere experienced by people living with bipolar disorder. These structures include stereotypes, norms, types of power distribution, communication methods, discriminative categories and labels, health care system, equality law, welfare system, and job status
9	Sarısoy et al. 2013	Turkey	Cross-sectional study	Available sampling	228 volunteers were included, 119 patients with bipolar disorder and 109 with schizophrenia	Rosenberg Self-Esteem Scale (RSES)	Internal stigma is seen in one in five people living with bipolar disorders, and anxiety or fear of communicating with others was more common in people living with bipolar disorders with internal stigma than in those without internal stigma
10	Cerit et al., 2012	Turkey	Cross-sectional study	Available sampling	80 bipolar patients	Oslo-3 Social Support Scale	The three predictors of people living with bipolar disorders’ performance were depression severity, perceived social support, and internal labeling, respectively
11	Thome et al., 2012	Brazil	Cross-sectional study	Available sampling	60 bipolar patients	Functioning Assessment Short Test (FAST) Stigma Experiences Scale (SES) Stigma Impact Scale (SIS)	The presence of depression symptoms and the age of treatment onset and diagnosis were also identified as predictors of internal stigma
12	Sadeghi et al. 2003	Iran	Cross-sectional study	Available sampling	100 bipolar patients 100 schizophrenia patients 100 MDD patients	A self—administered 34 items questionnaire	Significant proportion of the psychiatric patients families suffer from stigmatization which in turn makes them feel ashamed of having such a patient. The type of psychiatric disorder and some of the demographic characteristics play a major role in this regard and significant relationship was observed between the duration of the disorder, number of hospitalization and rate of being humiliated

### Consequences of sigma in people living with bipolar disorders and their families

Stigma has considerable consequences for people living with bipolar disorders and their families and causes them to suffer from severe psychological distress in addition to the pain and agony inflicted by the disease. In Table 5, it is mentioned information on articles that have examined the consequences of stigma in people living with bipolar disorders and their families and a summary of the findings of these articles is reported.

**Table 5:** Information on articles that have examined the consequences of stigma in people living with bipolar disorders and their families

	Author, Year	Location of study	Study type	Sampling method	Sample size	Tools	Finding
1	Latifian et al. 2022a, b, c	Iran	Qualitative Method (content analysis)	Purposive sampling	20 family members of bipolar patients 4 bipolar patients 3 mental health professional	Qualitative interviews	Social deprivation, emotional and sentimental excitement, objective and behavioral reflections, family solidarity threat, and separation from society were as the most important consequences of stigma in the family of bipolar patients
2	Kumar et al., 2020	India	Cross-sectional study	Random sampling	100 patients (50 patients with schizophrenia and 50 patients with bipolar disorder)	Quality of Life scale (WHO)	One of the most important consequences of stigma was disease relapse, facilitated by factors such as witnessing unfair behaviors by family members, the community, or the workplace
3	Quenneville et al., 2020	Switzerland	Comparative study	Available sampling	244 French-speaking patients (39 patients had a diagnosis of BPD, 136 had ADHD and 69 had BD)	The Brief Illness Perception Questionnaire	An increasing score of internal stigma influences quality of life and job performance in people living with bipolar disorders
4	Bhattacharyy a et al., 2019	India	Cross-sectional study	Available sampling	62 bipolar patients	Mental Illness (ISMI) scale Rosenberg Self‑esteem Rating Scale (RSES) World Health Organization (WHO) QOL‑BREF	Stigma causes people living with bipolar disorders to experience low self-esteem and a poor quality of life
5	Au et al., 2019	China	Cross-sectional study	Random sampling	115 bipolar patients with	Self-Stigma of Mental Illness Scale (C-SSMIS) Functional Assessment Short Test (FAST) Stigma Coping Orientation Scale (SCOS)	People living with bipolar disorders employ strategies such as avoiding others and social isolation to deal with stigma. Decreased self-esteem is the last phase in self-stigmatization and the most important phase in determining psychosocial performance
6	Post et al. 2018	Austria	Comparative study	Purposive sampling	60 outpatients with bipolar I disorder and 77 healthy subjects from the general community	Views on Manic Depression Questionnaire	Quality of life was significantly associated with resilience, internal stigma, and residual symptoms in people living with bipolar disorders. Even during recovery, people living with bipolar disorders experience a much poorer quality of life and lower resilience compared with healthy individuals
7	Grover et al. 2016	India	Cross-sectional study	Purposive sampling	185 bipolar patients	Bipolar Recovery Questionnaire	People living with bipolar disorders, internal stigma was seen in 28%, the experience of discrimination in 38.9%, experienced stigma after hiding from others in 28.6%, and social isolation in 28.6% of the patients. Regarding participation, about two-fifths of the patients revealed minimal activities. Finally, high levels of stigma were directly related to reduce mean life expectancy
8	Noack, Kirsten et al., 2016	Canada	Qualitative Method	Purposive sampling	29 bipolar patients	Qualitative interviews Rosenberg Self-esteem Rating Scale(RSES)	Stigma significantly decreases the participation of people living with bipolar disorders in society, making it difficult for them to acquire credible information about the disease, even via online resources
9	Sadighi et al., 2015	Iran	Cross-sectional study	Available sampling	126 bipolar patients	The Burden Assessment Schedule (BAS)	Stigma in people living with bipolar disorder has remarkable effects on the quality of life of these people and reduces their psychosocial performance and self-esteem
10	Karidi et al., 2015	Greece	Cross-sectional study	Random sampling	120 patients with schizophrenia and BD	Internalized Stigma of Mental Illness scale	Stigma itself led to social deprivation and poor performance in individuals, and the fact that psychiatric disorders have a direct and profound effect on self-stigma
11	Mileva et al. 2013	Canada	Cross-sectional study	Random sampling	392 bipolar patients (178 people from Argentina and 214 people from Canada)	Stigmatizing Experiences (ISE) Stigma Experiences Scale (SES) Stigma Impact Scale (SIS)	More than 50% of respondents believed that stigma had affected their life quality and reduced their self-esteem. For a better understanding of stigma in people living with bipolar disorders and their families, we must first know how they experience the phenomenon, and ISE is a valuable and reliable tool allowing us to reach this goal with high certainty in different cultures
12	Michalak et al., 2011	Canada	Qualitative Method	Purposive sampling	32 bipolar patients	Qualitative interviews Arts-based method (graphic recording)	Internal stigma could significantly affect self-management by people living with bipolar disorders. A reduction in self-management following internal stigma in these patients leads them to constantly regain their lost identities and roles in society
13	Vázquez et al. 2011	Three Latin American countries (Argentina, Brazil, and Colombia)	Cross-sectional study	Random sampling	241 bipolar patients (Argentina = 96, Brazil = 60, Colombia = 85)	Day’s Mental Illness Stigma Scale	A high perceived stigma score was directly and significantly correlated with a low-performance score
14	Brohan et al., 2011	13 European countries	Cross-sectional study	Available sampling	1182 people with bipolar disorder or depression	The Internalized Stigma of Mental Illness Scale (ISMI) The Boston University Empowerment Scale (BUES) The Perceived Devaluation and Discrimination Scale (PDD)	21.7%, 59.7%, and 71.6% of people living with bipolar disorders experienced self-stigma, resistance to stigma, and discrimination, respectively
15	Perlick et al. 2007	UK	Cross-sectional study	Random sampling	Caregivers of 500 people with bipolar disorder	Standardised Affective Disorder Evaluation Mini International Neuropsychiatric Interview The Clinical Monitoring Form	Caregivers' perceptions of stigma may negatively affect their mental health by reducing their coping effectiveness
16	Perlick et al. 2001	UK	Cross-sectional study	Available sampling	264 people with bipolar disorder	Schedule for Affective Disorders and Schizophrenia, Lifetime Version (SADS-L) The Brief Psychiatric Rating Scale (BPRS) The Social Adjustment Scale (SAS)	Concerns about the stigma associated with mental illness reported by patients during an acute phase of bipolar illness caused poorer social adjustment seven months later with individuals outside the patient's family

### 4- Effective interventions and strategies to reduce stigma in people living with bipolar disorders and their families

Numerous strategies and interventions have been suggested to cope with stigma and reduce its consequences in people living with bipolar disorders and their family members. Some of these strategies have been studied, and their positive outcomes in reducing stigma have been somehow illuminated. In Table 6, it is mentioned information on articles that have examined the effective interventions and strategies to reduce stigma in people living with bipolar disorders and their families and a summary of the findings of these articles is reported.

**Table 6:** Information on articles that have examined the effective interventions and strategies to reduce stigma in people living with bipolar disorders and their families

	Author, Year,	Location of study	Study type	Sampling method	Sample size	Tools	Finding
1	Latifian et al. 2022a, b, c	Iran	Quasi-experimental study	Random sampling	71 family members of bipolar patients	The Modified Version of the Internalized Stigma of Mental Illness Questionnaire The Opinion about Mental Illness (OMI) Questionnaire The provision of the psychoeducation package to the families of bipolar patients	Psychoeducation can be useful to reduce the internalized stigma of family members of bipolar patients and to increase their positive attitudes towards psychological disorders
2	Keshavarzpir et al., 2020	Iran	Quasi-experimental study	Random sampling	76 bipolar patients	Psychoeducation intervention Mental Illness scale stigma	Psychological education, as one of the supportive approaches to alleviate psychiatric problems, was reported to improve patients’ understanding of psychiatric disorders, which can positively affect self-esteem and the ability to manage stigma
3	Richardson et al., 2019	UK	Case series study	Random sampling	23 participants across 3 groups	Consultation method (World Café)	Cognitive-behavioral-based psychological education increased perceived improvement in people living with bipolar disorders and delayed the recurrence of mood disorders, highlighting the importance of factors such as identity, hope, optimism regarding the future, and empowerment. Overall, the intervention employed in this study was shown to reduce stigma and improve the quality of life in people living with bipolar disorders
4	Nilsson et al. 2016	Denmark	Naturalistic cohort study	Purposive sampling	50 remitted BD patients	Internalized Stigma of Mental Illness scale (ISMI)	Psychoeducation and affective temperaments were identified as factors in improving perceived stigma in bipolar patients
5	Hawke et al. 2014	Canada	Quasi-experimental study	Random sampling	137 participants ( health-care service providers, university students in a health-care-related course, people with BD and their friends and family members and the general public)	Oslo-3 Social Support Scale	In this study, a movie-based intervention was used and its significant impacts on reducing stigma in caregivers. Students also showed remarkable improvements in their tendency not to reduce the social distance from people living with bipolar disorders; which was also remarkable in the general public
6	Michalak et al., 2014	Canada	Mixed-Method	Purposive sampling	Quantitative phase: 80 bipolar patients 84 health care providers Qualitative phase: 14 bipolar patients 19 health care providers	Day’s Mental Illness Stigma Scale Mental Illness: Clinicians’ Attitudes Scale Version 4 Internalized Stigma of Mental Illness scale Performance evaluation scale Qualitative interviews	In this study, integrated methods were employed in the form of designing and performing theater to improve stigmatizing attitudes and the results showed that Caregivers, people living with bipolar disorders, and their families experienced significant improvements in their labeling attitudes immediately after performing the program

## Discussion

This study aimed to examining the experience of stigma and evaluating predictors, consequences and strategies to combat stigma in people with bipolar disorder and their families. Our findings suggest that people living with bipolar disorders and their families experience different levels of stigma, whose consequences, in general, include feelings of disrespect, disregard, and discrimination in society. To cope with this phenomenon, families often choose social isolation and withdrawal. They deprive themselves and the patient of receiving treatment by hiding the ill family member and delaying seeking treatment. Social stigma is the most devastating when the bipolar patients and their families accept it and internalized the negative views of the community, a phenomenon called internalized or emotional stigma. This is consistent with the findings of Bruni, Sharma, Wong, Bassirnia, Park, Grover, Ando and Leporil (Bruni et al. 2018; Sharma et al., 2017; Wong et al. 2009; Bassirnia et al. 2015; Park et al., 2014; Latifian et al. 2022a, b, c; Grover et al. 2019; Ando et al. 2013; Richard-Lepouriel et al. 2021).

The results of Ring and Thibodeau’s studies also showed that, people with mental health problems and their families experience stigma in various degrees. The experience of stigma in these people has led to inner turmoil in response to the stigma of others and weakening of family status among relatives and acquaintances. In addition, examining the experience of stigma in patients with schizophrenia and their families also showed that these people have problems in interpersonal interaction, structural discrimination, public images of mental illness and access to social roles, which all confirm the findings of this study (Ring et al., 2019; Thibodeau et al., 2017).

The analysis of available studies revealed that many factors could effectively predict stigma in people living with bipolar disorders and their families, including social and cultural structures, inefficient welfare system, low education, unemployment or lack of a suitable job, low self-esteem, poor communication skills, lack of intimate relationships with others, lack of being understood by others, poor social support, collectivist cultures, young age at disease onset, recurrent hospitalizations, gender, disease severity, disease duration, discriminative labels, lack of information/knowledge, generalization, banalization and reaction induced by public stigma in the general population, or among professionals such as health care professionals and employers. This finding was consistent with the results of Bonnington, Clemente, Favre, Sadeghi, Engidaw, Shumet, Thome, Cerit, Sarisoy, Howland, Ellison, Nilsson and Pal (Bonnington & Rose 2014; Clemente et al. 2017; Favre et al. 2022; Sadeghi et al. 2003; Engidaw et al. 2020; Shumet et al. 2021; Thomé et al. 2012; Cerit et al. 2012; Sarısoy et al. 2013; Howland et al. 2016; Ellison et al. 2015; Nilsson et al. 2016; Pal 2020).

In addition, the results of Bruni and Khaleghi’s studies also showed that in patients with psychiatric disorders, some personality traits may be accompanied by better empathic and communication skills, and may have a protective role against stigma. In these studies, violence, lack of knowledge and negative attitudes were introduced as predictors of stigma in psychiatric disorders (Bruni et al. 2018; Khaleghi et al. 2018). Moreover, the results of Świtaj’s study showed that in schizophrenia, saying offensive things about the mentally ill (69%), viewing unfavorably by others (63%) and treating them as less competent people (59%) identified as predictors of experience stigma in these patients and their families (Świtaj et al. 2009).

Studies have pointed out several consequences of stigma in these individuals, such as reduced participation, social deprivation, threats to mental health and reduced social adjustment emotional and sentimental excitement, objective and behavioral reflections, family solidarity threat, social exclusion, social isolation, restriction in social functions such as job performance and education, low self-esteem, poor quality of life, the prolongation of the treatment course, disease recurrence, hiding the disease, experiencing discrimination and injustice, and finally reduced life expectancy and resilience. These results were in line with previous studies of Bhattacharyya, Pal, Noak, Latifian, Perlick, Kumar, Sadighi, Au, Vazquez, Karidi, Post, Quenneville, Grover, Brohan and Mileva (Bhattacharyya et al. 2019; Pal 2020; Noack et al. 2016; Latifian et al. 2022a, b, c; Perlick et al. 2001, 2007; Kumar et al. 2020; Sadighi et al. 2015; Au et al. 2019; Vázquez et al. 2011; Karidi et al. 2015; Post et al. 2018; Quenneville et al. 2020; Grover et al. 2016; Brohan et al. 2011; Mileva et al. 2013).

The findings of Violeau and Klein's studies are also consistent with the results of this research. Because these studies have shown that the fear of being stigmatized or socially sanctioned and disgraced governs many aspects of human behavior. In many cases, the fear of stigma does not result in actual behavior change but rather leads individuals to simply hide certain behaviors or actions (for example, smoking in secrecy). When labeled as “schizophrenic,” patients feel a change in the way they are treated. This label affects the way patients interact with healthcare services as well as the world, since the illness becomes the central aspect of the patient’s identity. The impact of stigma on patient self-esteem should also be mentioned, since it is associated with a higher risk of depression and suicide (Violeau et al. 2020; Klein et al. 2021).

Several interventions have been suggested to alleviate stigma and its consequences in people living with bipolar disorders and their family members. The positive outcomes of these interventions included boosting public awareness and amending public attitudes toward bipolar disorder. Some of them included awareness, using alternative non-pharmaceutical therapies, enhancing self-esteem, using the ISE tool to identify patients’ experiences, psychological education and cognitive-behavioral interventions aiming to increase patients’ perceived recovery and sense of disease control. These results were in parallel with those of Michalak, Keshavarzpirpir, Richardson, Mileva, Post, Latifian, Nillson and Hawke (Michalak et al. 2011, Keshavarzpir et al. 2020; Richardson & White 2019; Mileva et al. 2013; Post et al. 2018; Latifian et al. 2022a, b, c; Nillson et al., 2016; Hawke et al. 2014).

Other evidence also suggests that health provider training can improve stigmatizing attitudes and that interventions combining positive messages of recovery potential with biological etiology will be most impactful to reduce stigma. Anti-stigma interventions designed specifically for health providers, family members, criminal justice personnel, and law enforcement seem particularly beneficial, given these sources of stigma (Wood et al. 2016). In addition, an understanding of the experience of family stigma can lead to the development of supportive strategies to manage this problem among caregivers of patients with psychiatry disorders. Mental health professionals can support caregivers by offering them opportunities to discuss how stigma is disrupting their caregiving roles. They can also support the caregivers in negotiating the experienced social and emotional distress and when necessary, refer them to the other members of healthcare teams (Ring et al., 2019).

## Limitations

The limitations of this study are the possibility of not including in-press articles, exclusion of non-English articles, the lack of possibility for searching in a number of other databases, and the possibility of the non-retrieval of all related studies using the combinations of the utilized keywords. So, to acquire all related articles, in addition to searching using a combination of syntax, authors also searched a considerable number of retrieved articles manually. Also, due to insufficient time and the compulsion to do a systematic review as a prerequisite for preparing a doctoral thesis, the protocol of this study was not registered.

## Conclusion

The results showed that stigma hurdles the treatment of people with bipolar disorder due to labeling, followed by hiding the disorder by families and delay in seeking treatment. Misconceptions such as considering these people dangerous and unpredictable and regarding families as culprits and irresponsible are present in society and workplace, educational settings, health care system, judiciary system, and even in the family. Therefore, it is necessary to take necessary measures to normalize bipolar disorder at the community level so that the general public becomes aware of its nature and understands stigma towards people with mental disorders. The findings of this study can provide useful information about stigma in people living with bipolar disorder, which can be used for mental health policymaking at the macro level, as well as by health care providers, the general public, and families at the micro level.

## Data Availability

The data that support the findings of this study are available.
